# Tumor physiological changes during hypofractionated stereotactic body radiation therapy assessed using multi-parametric magnetic resonance imaging

**DOI:** 10.18632/oncotarget.16395

**Published:** 2017-03-21

**Authors:** Heling Zhou, Zhang Zhang, Rebecca Denney, Jessica S Williams, Jeni Gerberich, Strahinja Stojadinovic, Debabrata Saha, John M Shelton, Ralph P Mason

**Affiliations:** ^1^ Department of Radiology, University of Texas Southwestern Medical Center, Dallas, TX 75390, United States; ^2^ Department of Radiation Oncology, University of Texas Southwestern Medical Center, Dallas, TX 75390, United States; ^3^ Department of Internal Medicine, University of Texas Southwestern Medical Center, Dallas, TX 75390, United States

**Keywords:** hypofractionated stereotactic body radiation therapy (SBRT), oxygen-sensitive MRI, blood oxygen level dependent (BOLD), dynamic contrast enhanced (DCE), treatment response

## Abstract

Radiation therapy is a primary treatment for non-resectable lung cancer and hypoxia is thought to influence tumor response. Hypoxia is expected to be particularly relevant to the evolving new radiation treatment scheme of hypofractionated stereotactic body radiation therapy (SBRT). As such, we sought to develop non-invasive tools to assess tumor pathophysiology and response to irradiation. We applied blood oxygen level dependent (BOLD) and tissue oxygen level dependent (TOLD) MRI, together with dynamic contrast enhanced (DCE) MRI to explore the longitudinal effects of SBRT on tumor oxygenation and vascular perfusion using A549 human lung cancer xenografts in a subcutaneous rat model. Intra-tumor heterogeneity was seen on multi-parametric maps, especially in BOLD, T_2_* and DCE. At baseline, most tumors showed a positive BOLD signal response (%ΔSI) and increased T_2_* in response to oxygen breathing challenge, indicating increased vascular oxygenation. Control tumors showed similar response 24 hours and 1 week later. Twenty-four hours after a single dose of 12 Gy, the irradiated tumors showed a significantly decreased T_2_* (-2.9±4.2 ms) and further decrease was observed (-4.0±6.0 ms) after 1 week, suggesting impaired vascular oxygenation. DCE revealed tumor heterogeneity, but showed minimal changes following irradiation. Rats were cured of the primary tumors by 3×12 Gy, providing long term survival, though with ultimate metastatic recurrence.

## INTRODUCTION

Lung cancer is the second most common non-skin cancer in both men and women and is the leading cause of cancer related death [1]. The majority of lung cancers are non-small cell (NSCLC) [2] and primary treatment options are surgery, radiation and chemotherapy. Early stage NSCLC patients, who can tolerate surgical resection have approximately 60-80% survival rate over 5 years [3]. For those patients ineligible for surgery, the standard of care is radiation therapy, which historically results in a much lower 5-year overall survival (6-30%) [4]. Conventional fractionated radiation therapy (CFRT) consists of 30 small daily fractions (typically, 2 Gy/fraction). However, recent clinical trials indicate that hypofractionated stereotactic body radiation therapy (SBRT) shows significantly improved response [5, 6]. The typical regimen (3 × 12 to 16 Gy) is delivered in 1½ to 2 weeks [6]. With fewer patient visits, customized treatment planning based on individualized tumor characteristics has become a possibility, implying the opportunity for precision medicine.

Hypoxia is recognized to play an important role in radiation resistance [7–9]. Historical evidence suggests that reoxygenation occurs during CFRT, but SBRT offers less opportunity [10]. Initial hypoxia and temporal changes could strongly influence radiation response [11]. To investigate tumor oxygenation and changes in response to high dose irradiation, we investigated the A549 tumor implanted subcutaneously as a xenograft in nude rats. The A549 is a popular tumor research model of human disease, but historically most investigations were performed in cell culture or in xenografts implanted in immune compromised mice [12–17]. *In vitro* studies indicate that A549 cells are relatively radioresistant with several reports over the years consistently indicating about 80% cell death at 6 Gy [17–20].

A549 tumor is reported to be very hypoxic with 80% of measurements showing pO_2_<2.5 Torr using polarographic oxygen electrodes for subcutaneous tumors in mice [21]. Substantial uptake of bioreductively activated nitroimidazoles has revealed hypoxia based on the SPECT agent ^99m^Tc-HL91 (Prognox) [21], the PET reporter fluoroazomycin arabinoside (F-18 FAZA) [22] and immunohistochemistry [23]. Hypoxia was readily modulated with hyperoxic gas breathing (oxygen or carbogen plus nicotinamide) [21, 22]. Several irradiation protocols have been reported indicating significant tumor growth delay. A single dose of 5 Gy caused tumor growth delay, but growth continued (50% over 14 days) [19]. Even 2×20 Gy caused only 20 day growth delay [24]. Several reports indicate that tumor hypoxia, perfusion and vascular permeability are altered following high dose irradiation to various tumor types [25–30] including A549 in mice [23].

Non-invasive measurements of tumor hypoxia would be particularly attractive for tumor progression and treatment planning. In this study, we applied two oxygen enhanced MRI techniques blood oxygen level dependent (BOLD) [31, 32] and tissue oxygen level dependent (TOLD) [33–35] MRI to assess vascular oxygenation and tissue oxygenation, respectively. It is reported that tumor BOLD and TOLD responses to oxygen breathing challenge can provide information on tumor oxygenation [36–39], which could be easily implemented into clinical practice [36, 40–42]. In addition, we applied DCE (Dynamic Contrast Enhanced) MRI to assess the vascular perfusion and tumor pathophysiology. We used these MRI techniques to evaluate the acute and chronic sequelae of high dose irradiation at 24 hrs and 1 week, as part of a three x 12 Gy irradiation protocol of subcutaneous A549 human lung tumor xenografts implanted in nude rats.

## RESULTS

**Figure 1:**
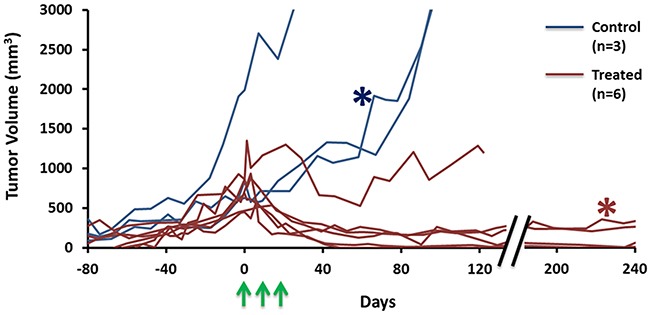
Hypofractionated SBRT caused tumor growth delay A549 tumor growth curves for nine rats (Blue: control, n=3; Red: treatment, n=6). Tumors initially showed expected exponential growth. Three doses of 12 Gy irradiation (green arrows) caused effective control and shrinkage in most cases. The representative tumors used in Figures 2 and 3 are marked with red and blue *, respectively.

The take rate of A549 tumors implanted subcutaneously in nude rats was sub optimal. With whole body irradiation (3 Gy) 48 hrs prior to implantation 27 tumors developed, but 10 regressed spontaneously. The remaining 17 showed somewhat variable growth rates with volume doubling times in the range 16 to 36 days and 13 were included in this study. Rats were divided into three groups: Group 1 served as control (n=3; tumor volume = 1,200±700 mm^3^, ranging from 600 to 2,000 mm^3^) received no irradiation and were sacrificed once the tumor volume exceeded 3,000 mm^3^, which occurred within 200 days post implantation (Figure 1). A fourth control tumor was used for baseline histology. Tumors in Group 2 (n=6; 700±200 mm^3^, ranging from 450 to 950 mm^3^) breathed oxygen 15 minutes before and during irradiation (12 Gy x three fractions, one week interval between each treatment) and all exhibited effective tumor growth control (Figure 1). No recurrence of primary tumors was observed. One tumor did remain at relatively constant volume, while all other treated tumors essentially disappeared, but all rats were sacrificed between 122 and 376 days post irradiation due to development of metastases (in pelvis and lung). Metastases were confirmed by DNA fingerprinting and comparison with reference to ATCC. Group 3 (n = 3; 800±200 mm^3^, ranging from 600 to 1,000 mm^3^) breathed oxygen 15 minutes before and during irradiation (12 Gy x one fraction only) and were sacrificed 24 hours later for histology.

**Figure 2:**
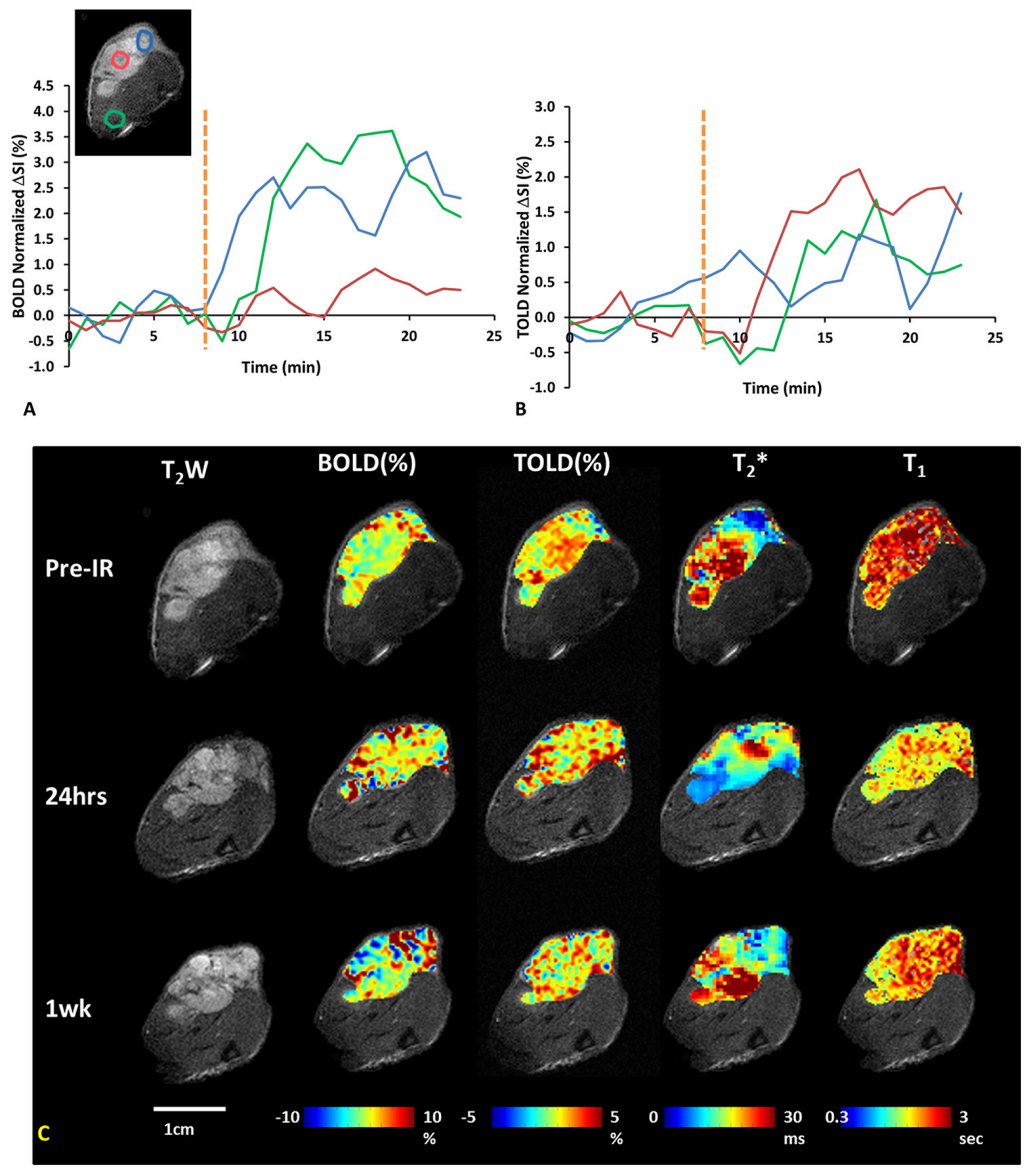
Oxygen sensitive MRI of a representative A549 lung tumor with respect to irradiation **(A)** ΔSI curves showing BOLD, and **(B)** TOLD responses for three ROIs indicated on T_2_w-image (blue tumor periphery, red tumor center and green adjacent muscle). Yellow dotted line indicates transition from air to oxygen breathing. **(C)** T_2_w-images and overlaid parametric maps showing %ΔSI of BOLD and TOLD, T_2_* and T_1_ maps (left to right) of tumor (0.5 cm^3^) before, 24 hours and one week after 1^st^ dose of radiation (12 Gy).

**Figure 3:**
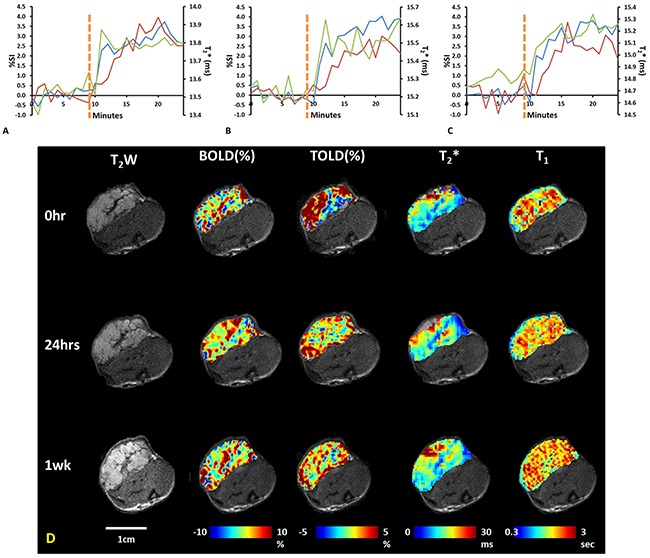
Oxygen sensitive MRI of a representative control A549 lung tumor Upper graphs: BOLD (blue), TOLD (red) and T_2_* (green) curves show response to oxygen breathing challenge of a control tumor at three time points: **(A)** baseline, **(B)** 24 hours, and **(C)** 1 week. Yellow dotted line indicates transition from air to oxygen breathing. **(D)** T_2_w images of tumor (0.7 cm^3^) and overlaid parametric response maps showing %ΔSI of BOLD and TOLD, T_2_* map and T_1_ map (left to right) at baseline, 24 hours and one week.

Oxygen sensitive MRI (T_2_* and T_1_ maps) showed distinct heterogeneity at baseline, while rats breathed air (Figures 2 and 3). The respiratory rate was typically 30-40 breaths per minute and SAO_2_ (arterial oxygen saturation) increased from 89% during air breathing to 97% with oxygen breathing (p<0.001). Rectal temperature was successfully measured in 33 of 35 studies and showed distinct stability with only minor fluctuations during a typical scan of 25 mins (maximum temperature changes 0.34±0.25°C, ranging from 0 to 0.9°C, n = 33). Moreover, the temperature fluctuations did not coincide with TOLD MRI responses to oxygen challenge, indicating little effect of temperature on T_1_ in this experimental setting. In response to oxygen breathing challenge, most tumors showed a positive BOLD signal response at baseline, which was significant (p<0.05) for all three tumors in Group 1 and seven of nine tumors in Groups 2+3. Observed responses for the groups were BOLD ΔSI (mean ±SD) = 3.0±0.5% for Control Group 1 (n=3) and 1.5±1.6% for Groups 2+3 (n=9) and a slight increase in T_2_* indicating increased vascular oxygenation (ΔT_2_* = 0.4±0.1 ms for Group 1 and 0.1±0.4 ms for Group 2+3), but due to the range of values the changes were not significant for the groups. Heterogeneity in enhancement was apparent, notably with greater response in the tumor periphery, particularly for BOLD and T_2_* (Figures 2 and 3). TOLD response was typically delayed somewhat compared with BOLD and T_2_* (Figure 3A). A significant (p<0.05) TOLD response (%ΔSI) to oxygen was observed for two of three tumors in Group 1 and seven of nine tumors in Group 2+3 prior to radiation.

Twenty-four hours later, the BOLD signal and T_2_* response to oxygen breathing challenge were significant (p<0.05) for each individual control tumor (Group 1; n=3). Likewise, the BOLD signal and T_2_* response to oxygen breathing challenge were significant (p<0.05) for each individual tumor 24 hours after radiation (Groups 2 and 3 (n = 9)). Most of the of TOLD ΔSI responses to oxygen were also significant (p<0.05; for all three tumors in Group 1 and seven out of nine tumors in Group 2+3). BOLD ΔSI (mean ±SD) = 2.8±0.8% for Control Group 1 (n=3) and -1.4±6.1% for Groups 2+3 (n=9) and ΔT_2_* = 0.4±0.2 ms for Group 1 and 0.3±0.4 ms for Group 2+3.

**Figure 4:**
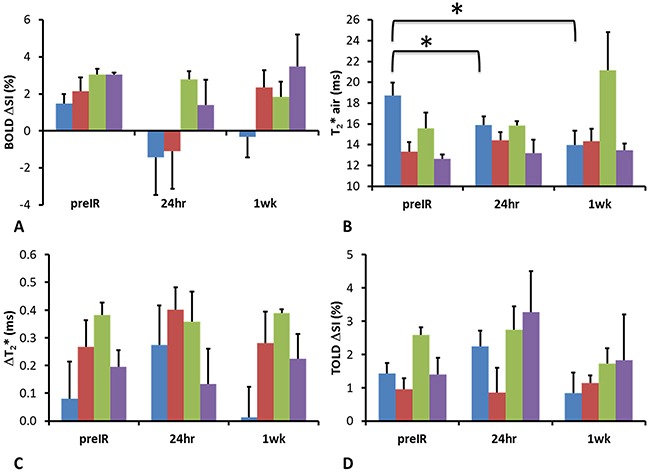
Summary of changes of oxygen sensitive parameters for irradiated group Graphs showing mean and standard errors of the mean pre IR, 24 hours and 1 week post radiation for tumors and adjacent muscle of the irradiated (n = 9 for preIR and 24 hours; n = 6 for 1 week) and control rats (n = 3). Irradiated tumor (blue), adjacent muscle (red) control tumor (green) and muscle (purple).* p<0.05 based on paired t-test.

Twenty-four hours after the first dose of radiation, tumors (n= 9) showed a significantly decreased T_2_* (-2.9±4.2 ms; p=0.045, one-tail paired Student's t-test compared to baseline) (Figure 4). T_2_* decreased further after 1 week (-4.0±6.0 ms; p=0.035, one-tail paired Student's t-test compared to baseline, n=6), implying decreased oxygenation (Figure 4). By comparison control tumors showed a similar BOLD response at 24 hours, which remained essentially unchanged 1 week later (Figure 3). Data are summarized in Figure 4. Adjacent thigh muscle showed no significant changes over the period of 1 week for irradiated or control rats (p=0.15 and 0.39, respectively).

A moderate positive correlation was observed between BOLD and TOLD signal responses at baseline (R^2^ = 0.47 for all tumors (Group 1, 2 and 3; n=12) and R^2^ = 0.40 for the subset of tumors subsequently irradiated (Group 2 and 3; n=9)). However, no correlation was found 24 hours after radiation (R^2^=0.03; Group 2 and 3; n=9). A correlation was observed one week later (Group 2; n=6; R^2^ = 0.64). Similarly, BOLD %ΔSI and ΔT_2_* showed good correlation at baseline and one week after radiation (R^2^ = 0.71 and 0.85 respectively), but not at 24 hours after radiation (R^2^=0.03).

**Figure 5:**
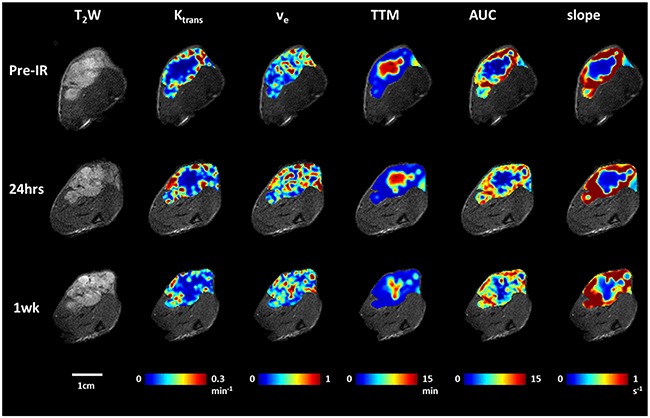
DCE parametric maps of the representative tumor pre, 24 hours and 1 week after first dose of radiation treatment T_2_-weighted images and overlaid parametric response maps showing, K^trans^, v_e_, TTM, AUC and slope (left to right) of the same tumor as Figure 2 pre, 24 hours and one week after first radiation therapy. Distinct heterogeneity is observed for all the maps. No obvious changes were observed following radiation compared to baseline.

**Figure 6:**
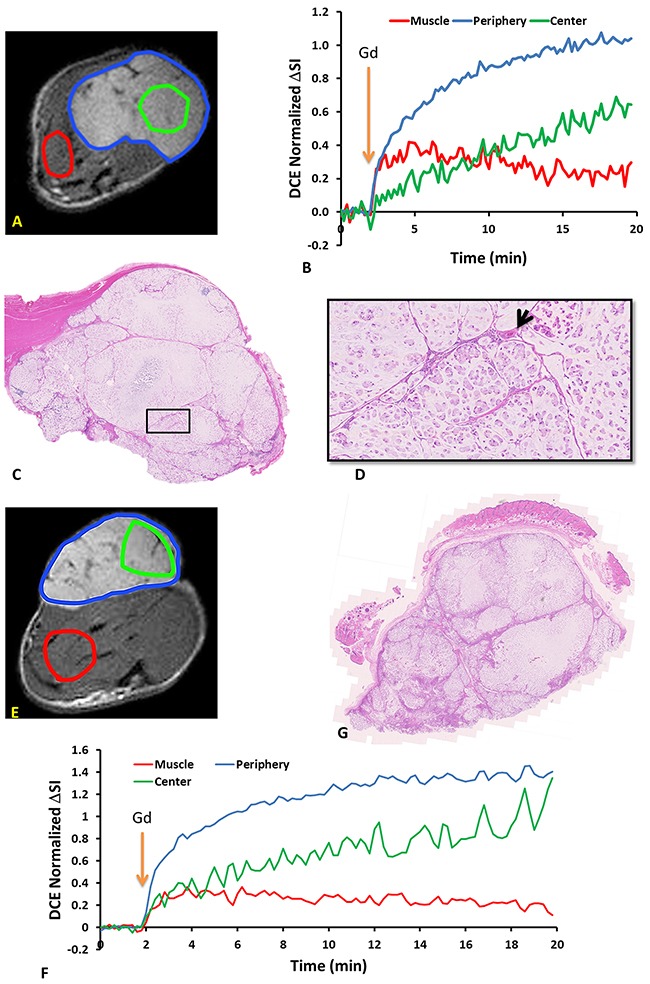
DCE dynamic curves and H&E staining revealed intra-tumor heterogeneity in both control and irradiated subcutaneous A549 human lung tumor xenografts Upper panel: control tumor **(A)** ROIs of tumor periphery, tumor center and muscle overlaid on T_2_w anatomical image of subcutaneous A549 human lung tumor xenograft. **(B)** DCE signal intensity curves of tumor periphery (blue region excluding green), tumor center (green) and thigh muscle (red) regions normalized to their mean baseline. Orange arrow indicates the time of Gd infusion. **(C)** H&E stained section of the same tumor. **(D)** Expanded region (black box). Arrow indicates a capillary in the connective tissue. Lower panel: tumor 24 hours after single dose of 12 Gy. **(E)** T_2_w MRI showing ROIs. **(F)** corresponding DCE signal intensity curves. **(G)** H&E stained section.

No obvious changes were observed in the DCE parametric maps of irradiated (Figure 5) or control tumors (Supplementary Figure 1). At baseline mean K*^trans^* = 0.14±0.04 min^-1^ and v_e_ = 0.40±0.05 for Group 1 and K*^trans^* = 0.13±0.05 min^-1^ and v_e_ = 0.44±0.06 for Groups 2 + 3. In general, there was a lack of correlation between oxygen sensitive and DCE parameters, but four distinct correlations were observed prior to irradiation, primarily with respect to v_e_ (Supplementary Figure 2). Baseline T_2_* was related to maximum signal observed following Gadovist infusion (R^2^=0.45). Correlations were observed between BOLD signal response, ΔT_1_, ΔT_2_* and v_e_ with R^2^= 0.60, 0.64, 0.37 respectively (Supplementary Figure 2 Graphs B-D). Twenty four hours after irradiation no correlations remained with R^2^< 0.26 in each case. The boundaries of the multi-nodular tumor were obvious on the functional maps both before and after radiation. DCE curves showed distinctly different patterns for tumor periphery and center of control (Figure 6A, 6B) and irradiated tumors (Figure 6E, 6F). H&E staining (Figure 6C, 6G) confirmed the multi-nodular structure observed in multi-parametric MRI maps. The tumor appeared to be rather avascular with most blood vessels located within the connective tissue between the pockets of tumor cells (Figure 6D). Structure appeared quite similar in control and irradiated tumors (Figure 6C, 6G).

**Figure 7:**
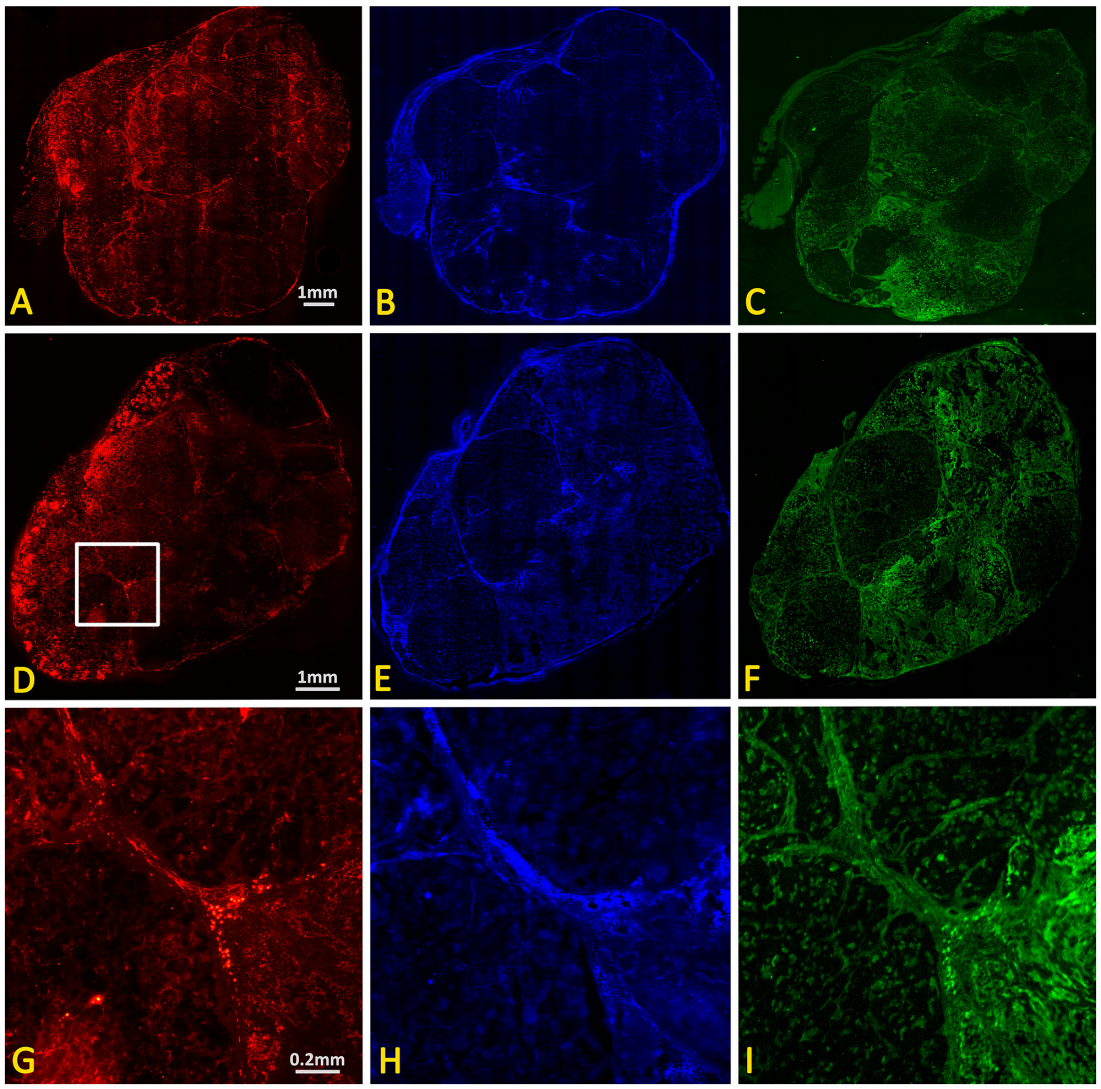
IHC of control and irradiated subcutaneous A549 human lung tumor xenografts Upper panel: Contiguous sections showing control tumor **(A)** vasculature based on CD31 immunohistochemistry, **(B)** tumor perfusion based on Hoechst 33342 distribution, and **(C)** hypoxia based on immunohistochemistry for perfused pimonidazole. Lower panel: **(D-F)** corresponding whole mount sections from tumor 24 hours after single dose of 12 Gy. Magnified sections (white box in D) are shown in **(G, H)** and **(I)**.

Immunohistochemistry of representative control and irradiated tumors confirmed heterogeneity with extensive non-perfused, essentially avascular, regions (Figure 7). Notably, pimonidazole uptake, which indicates hypoxia, was greater in vascular regions, suggesting failure to reach non-perfused regions.

## DISCUSSION

The majority of A549 human NSCLC xenografts grew in the thigh of nude rats following whole body irradiation and implantation of 3×10^6^ cells. MRI revealed very heterogeneous multinodular structure. DCE MRI indicated little change over a period of one week for control tumors or following irradiation. Most oxygen sensitive parameters showed little change, but T_2_* was significantly decreased following irradiation after both one and 7 days suggesting reduced vascular oxygenation. The regimen of 3×12 Gy halted tumor growth, and most tumors shrank. No recurrence was observed at the primary tumor site over the period of study, but rats required sacrifice due to development of aggressive metastases after 122 to 376 days.

The A549 tumor is reported to be radioresistant [18], but growth was essentially halted by a single dose of 12 Gy irradiation in rats breathing oxygen. Following a second dose of 12 Gy, all tumors had decreased in size and following three doses, 5 of 6 tumors essentially disappeared, while one remained as a constant 1 cm^3^ nodule. No recurrence was observed at the primary site at the time of sacrifice. In each case, the rat was sacrificed for ethical reasons, since a large mass developed in the peritoneum. Additional metastases were found in lung through necropsy. DNA was extracted from the tumor tissue and verified by DNA fingerprinting, where the sample fingerprints were compared against the database of reference fingerprints collected from ATCC and other core facility resources confirming the metastases to be A549.

MRI revealed distinct heterogeneity in tumor anatomical structure. The relaxation parameter maps also showed wide heterogeneity with regional (voxel) T_1_ values ranging from 1.2 to 5.8 s (mean T_1_ = 2.6±0.6 s) in a typical tumor (Figure 2) and mean baseline tumor T_1_ in the range of 1.5 to 2.8 s (population mean = 2.3 ± 0.4 s; n=8). This is similar to measurements in Dunning Prostate R3327-AT1 tumors and 9L gliomas reported at 4.7 Tesla [34, 38]. Spin lattice relaxation has been reported for A549 tumors, but at substantially different magnetic fields, which is known to alter relaxation (9.4 T [19] and 11.75 T [43]). T_2_* showed a typical range 0.8 to 56.2 ms (mean = 19.7±10.2 ms) in an A549 tumor with a range of mean tumor values = 12.7 to 23.7 ms (population mean = 18.7±3.8 ms; n=9). Again, this was similar to literature reports of R_2_* mean = 44 to 66 ms for AT1 and HI tumors [38]. The semi-quantitative BOLD response was quite similar to previous observations in relatively hypoxic large Dunning Prostate R3327-AT1 tumors [38, 39]. All these oxygen sensitive parameters were quite consistent in the control tumors when repeated after one and seven days. As expected, BOLD and T_2_* responses to oxygen breathing challenge were essentially synchronous, since the BOLD effect is derived from the third echo of the T_2_* decay curve (Figure 3). The TOLD response was, however, delayed slightly compared to BOLD, since oxygen is initially delivered through the inflow of oxyhemoglobin and subsequent diffusion into the tissues.

In response to irradiation most parameters were essentially unchanged after 24 hours and one week. BOLD response decreased, but the change was not significant. The only significant change was in transverse relaxation time T_2_*, which was significantly decreased after both one and seven days suggesting increased relaxation and greater deoxyhemoglobin. This contrasts our recent observations in radiation resistant Dunning prostate R3327-AT1 tumors, where there was evidence for reoxygenation in some tumors based on pulse chase immunohistochemistry and oxygen enhanced MRI following 15 Gy radiation, which was associated with greater tumor growth delay (based on volume quadrupling time) following a second dose of 15 Gy radiation [30]. Several studies have shown previously that BOLD and/or TOLD responses correlated with pO_2_ measurements and tumor growth delay following radiation [30, 39, 44]. The utility of a TOLD (also referred to as Oxygen-enhanced) MRI response to oxygen breathing challenge to assess tumor hypoxia was thoroughly investigated and validated recently in subcutaneous 786-0 renal cancer xenografts in mice [35]. A strong correlation was observed between the fraction of tumor which failed to respond to oxygen breathing challenge (so-called Oxy-R fraction) and hypoxic fraction assessed using pimonidazole immunohistochemistry. Meanwhile, in SW-620 colorectal carcinoma there was no obvious correlation, until tumor sub regions were stratified according to perfusion status derived from DCE analysis, thus emphasizing the potential utility of combined DCE and oxygen enhanced MRI.

Recently, a quantitative MRI oximetry technique, MRI-based Oxygen Imaging (MOXI) [45] was proposed, whereby a pO_2_ value can be derived from a mathematical model with a combination of quantitative T_1_, T_2_ and diffusion measurement. Another oximetry technique, “mapping of oxygen by imaging lipid relaxation enhancement” (MOBILE) [46] compared ΔR_1_ responses in tissue water and lipid signals with ΔR_2_* of water as a function of oxygen breathing challenge to evaluate potential changes in tumor oxygenation. R_1_ lipid response was much greater than that of water and correlated strongly with pO_2_ assessed using a fiber optic probe or ESR reporter. Indeed, at 11.7 Tesla the response of the lipid at 4.0 ppm was about 11 times greater than water, whereas that at 1.3 ppm was about 2 fold. However, the approach is complicated by the need for water suppression and at lower magnet fields the 4 ppm lipid signal was not easily resolved from water. Lipid also exhibits relatively lower signal to noise ratio. Oddly, a follow up study examining relative R_1_ responses in 9L-glioma and rhabdomyosarcoma found smaller response for lipid R_1_ than water R_1_ [47]. This recent work indicated that only R_2_* had predictive value with respect to tumor irradiation and this applied to subcutaneous 9L glioma in rat, but not rhabdomyosarcoma.

Dynamic contrast enhanced MRI also showed distinct intra tumor heterogeneity (Figure 5, Supplementary Figure 1). At baseline, parameters indicated greater peripheral perfusion (*e.g*., TTM, AUC, slope), but with obvious well perfused inclusions, attributable to the vessels surrounding individual nodules. The control tumor indicated quite constant DCE over one week and no significant changes were found following irradiation. Mean K*^trans^* ranged from 0.04 to 0.22 min^-1^ in individual tumors with population mean 0.13±0.05 min^-1^ (Groups 1-3). By comparison Hallac *et al*. reported K*^trans^* =0.11 ±0.05 min^-1^ in AT1 tumors [48]. Mean v_e_ ranged from 0.35 to 0.58 for individual tumors with population mean 0.43±0.07 (Groups 1-3), whereas Hallac *et al*. reported 0.19 ±0.09 in AT1 tumors [48]. That study indicated a strong correlation between v_e_ and time to quadruple in volume following single high dose irradiation (30 Gy), but no similar conclusions can be drawn here, since none of the tumors recurred. The v_e_ values observed here were considerably larger than reported values [48]. This is not surprising given the difference in cell density between human lung cancer A549 and rat prostate cancer AT1. It is evident in H&E that the cell density of A549 tumor (Figure 6C) was much lower compared to the dense tumor in AT1 subcutaneous model [49, 50]. Others have reported heterogeneity in subcutaneous A549 tumors implanted in mice with much greater enhancement in tumor periphery than center [19, 23]. Lee *et al*. reported K*^trans^* = 0.0812±0.014 min^-1^ [19]. Pishko *et al*. [43] examined A549 tumors implanted in the brains of nude rats and reported R_1_ was in the range 0.45 to 0.54 ms^-1^ and K*^trans^* = 0.027 to 0.084 min^-1^. Differential peripheral versus central perfusion is also seen in human lung cancer: in a study of 16 patients using dynamic contrast enhanced CT during a course of 27 Gy over three weeks the tumor center showed much lower perfusion and little variation, while the periphery showed a peak increase in vascular volume and permeability after 18 Gy [51]. There was a general lack of correlation between BOLD, TOLD and DCE parameters. However, four distinct correlations were observed prior to irradiation (Supplementary Figure 2). The inverse correlation observed between BOLD signal response or ΔT_2_* and v_e_ is to be expected since high v_e_ (the fraction of extravascular extracellular space) is consistent with low cell density, necrosis and sparse vasculature.

As reported by others, generating A549 human tumor xenografts in nude rats is not straightforward and benefits from total body irradiation prior to tumor implantation [52, 53]. It has been suggested that residual innate immunity prevents tumor development and we found that whole body irradiation two days prior to implantation was crucial to facilitate growth. In the subcutaneous setting, we also found that A549-luc cells (stably transfected to express luciferase) did not implant and grow well, even after whole body irradiation, implying potential additional immunogenicity attributable to transgene expression (unpublished results). Orthotopic implantation was more successful and new approaches to implantation and development of single nodules have been reported recently [54, 55]. However, MRI is more challenging in the orthotopic lung setting and methods are under development. The initial time for tumor development was highly variable, but once tumors reached 500 mm^3^, they showed a typical volume doubling time of 27 days (Figure 1). Tumors were found to grow with multi nodular structure visible by MRI (*e.g*., Figure 2) and companion histology (Figure 6). The tumors also tended to infiltrate the muscle, as opposed to more normal subcutaneous growth observed with many other tumor types [39]. The tumors exhibited perfusion around the whole tumor and around the individual nodules, creating highly heterogeneous vasculature observed by dynamic contrast enhanced MRI (Figure 5, Supplementary Figure 1) and confirmed by histology (Figures 6 and 7).

The recent review of Park *et al*. indicated highly variable tumor responses with respect to irradiation [56]. Indeed, comparison of reports is difficult due to diverse sizes of different tumor sublines, growing at various locations, and with respect to different irradiation doses and timing schedules. There is evidence in patients with resectable lung cancer that hypoxic tumors were associated with poor prognosis following surgery [57]. We note that motion can lead to artifacts in MRI of lung tumors and that the tissue air boundaries make lung cancer challenging, but we have reported preliminary measurements of BOLD responses (both semi-quantitative changes in signal intensity and ΔR_2_*) in human patients [58].

This study showed reduced vascular oxygenation as indicated by the significant decrease in T_2_* compared to baseline one and 7 days after first fraction of 12 Gy, but no significant response in dynamic contrast enhanced MRI at these times. All rats were cured of their primary tumors with 3×12 Gy, but the vascular hypoxiation may become relevant for optimizing treatment interval in future studies.

## MATERIALS AND METHODS

### Cell preparation

A549 (wild type) human lung cancer cells (ATCC, Manassas, VA) were incubated in Dulbecco's modified Eagle's medium (DMEM) with 10% fetal bovine serum (FBS), 1% *L*-glutamine and 1% penicillin-streptomycin at 37 °C with 5% CO_2_. Once 80% confluence was reached, the cells were harvested, and suspended in serum-free medium.

### Tumor model

All animal procedures were approved by the Institutional Animal Care and Use Committee of the University of Texas Southwestern Medical Center in accordance with Federal, State, and Local laws and guidelines and consistent with Guidelines for the welfare and use of animals in cancer research [59]. Thirty anesthetized nude rats (8-10 week old female; T-cell-deficient, athymic homozygous nude rat; Charles River, NCI at Frederick, Frederick, MD) were pre-treated with whole body radiation (3 Gy) to achieve additional immunosuppression and 48 hours later A549 human lung cancer cells (3×10^6^, in 200 μl serum free medium with 50% Matrigel (Corning Inc., Corning, NY)) were implanted subcutaneously in the right hind thigh [52]. The tumor volume was measured by mechanical caliper every week to determine the tumor growth based on three orthogonal diameters (x*y*z*π/6). Tumors that did not initiate successfully or spontaneously regressed were excluded from the study.

### MRI

When the tumor volume reached 500-1,000 mm^3^, baseline MRI was performed using a horizontal bore 4.7-T magnet (Varian, Palo Alto, CA) with homebuilt 3.5 cm diameter single-turn solenoid volume coil [49]. Animals were anesthetized with isoflurane (1.5%) in air (1 L/min) and kept warm using a circulating warm water blanket. Animal body temperature and respiration were monitored with a small animal physiological monitoring system (Small Animal Instruments, Inc. Stony Brook, NY) throughout the experiment. A catheter was secured in the tail vein and kept *in situ*. T_1_ maps (spin echo; TE= 20 ms, TR= 0.1, 0.2, 0.3, 0.5, 0.7, 0.9, 1.5, 2.5, 3.5 s) were acquired during initial air breathing and with oxygen challenge prior to DCE. Interleaved BOLD (multi-echo gradient echo; TR = 150 ms, ten echo times from 6 to 69 ms, flip angle = 20° with acquisition time 38 seconds per map) and TOLD (gradient echo; TR/TE = 30/5 ms, flip angle = 45° with total acquisition time 3 seconds per dynamic) MRI were acquired with the intervention of an oxygen challenge (from air to 100% O_2_ after about 10 mins). With continued oxygen breathing, DCE (spin echo; TR/TE = 200/15 ms) was performed with IV injection of gadolinium contrast (0.1 mmol/kg body weight Gadovist, Schering, Berlin, Germany). MRI was performed on the day prior to, 24 hours and one week after the first SBRT treatment.

### Irradiation

Twelve rats were divided into three groups: Group 1 served as control (n=3, no irradiation). Group 2 (n=6) breathed oxygen 15 minutes before and during irradiation (12 Gy x three fractions, one week interval between each treatment). Group 3 (n = 3) breathed oxygen 15 minutes before and during irradiation (12 Gy x one fraction only), sacrificed 24 hours later for histology. For each fraction, the prescribed 12 Gy radiation dose was delivered with two equally weighted parallel-opposed anterior-posterior (AP) and posterior-anterior (PA) beams utilizing an image guided small animal x-ray irradiator (XRAD 225Cx, Precision X-Ray, Inc., North Branford, CT). An additional rat was used for control histology.

### Data analysis

Data were processed and statistical analysis was done using Matlab. Semi-quantitative percentage signal intensity changes (%ΔSI) of BOLD and TOLD and quantitative T_1_ and T_2_* maps were calculated. R^2^>0.8 was set as a threshold to ensure curve fitting reliability. Area under the curve (AUC), time-to-maximum (TTM) and slope were calculated from DCE. A reference tissue method was used for the quantitative analysis to obtain blood perfusion-vessel permeability product (K*^trans^*, unit min^-1^) and extravascular-extracellular volume fraction (v_e_) [60].

### Histology

Twenty-four hours after the first radiation dose, MRI was repeated for Group 3 rats. Following MRI, while rats continued to breathe oxygen, pimonidazole hydrochloride (Hypoxyprobe-1; NPI, Burlington, MA) was injected into the tail vein at a dose of 60 mg/kg. An additional control non-irradiated rat breathing oxygen received pimonidazole. Sixty minutes later, perfusion marker Hoechst 33342 (10 mg/kg, Life Technologies, Carlsbad, CA) was injected IV and the rat was sacrificed 1 minute later. Tumors were dissected, oriented transaxial to match MRI orientation, and flash-frozen in OCT freezing matrix (Sakura Finetek USA, Torrence, CA). Contiguous sections in the transaxial plane were obtained on a Leica CM3050S cryostat (8 μm, Leica Microsystems, Buffalo Grove, IL) for routine hematoxylin and eosin staining (H&E), immunohistochemical detection of vascular endothelium (CD31/PECAM) and pimonidazole hypoxia marker, as well as for direct visualization of Hoechst dye uptake.

In detail, sets of four serial cryostat sections were thaw-mounted onto silanated microscope slides, air-dried with laminar flow, and packaged for storage at -80 °C for later use. Subsequently, slides for immunohistochemistry were thawed for 30 minutes with laminar flow and immediately imaged without cover slips using illumination to detect Hoechst 33342. Following imaging for Hoechst dye, two separate and contiguous slides were subjected to differing optimized protocols for pimonidazole and CD31 immunohistochemistry. Slides intended for pimonidazole detection were readied for staining by fixation in refrigerated acetone, air-drying, and rehydration in phosphate buffered saline (PBS, pH 7.3), prior to permeabilization in 0.1% Tween. Pimonidazole slides were then rinsed free of surfactant permeabilizer in PBS, blocked with 3.5% normal mouse serum/0.5% bovine serum albumin (BSA), and probed for one-hour incubation with fluorescein-conjugated monoclonal mouse anti-pimonidazole primary antibody (1:60 in 0.5% BSA/PBS; clone 4.3.11.3; NPI). Slides were washed free of unbound primary in PBS and cover slipped for later imaging. Slides intended for CD31 detection were readied for staining by fixation in refrigerated 4% paraformaldehyde/PBS (4% PFA, pH 7.3), washing in PBS, permeabilization with 1x trypsin (10 mins at 37 °C), and an additional 5 mins post-fixation in 4% PFA. CD31 slides were then rinsed free of final fixative in PBS, blocked with 3% normal goat serum/1% BSA/PBS, and probed for a day-and-a-half (36-42 hrs at 4 °C) with monoclonal mouse anti-rat CD31 (1:10 in 1% BSA/PBS; clone TLD-3A12; BD Biosciences, San Diego, CA). CD31 slides were subsequently washed in PBS and bound primary antibody was detected by incubation with Cy3-conjugated goat anti-mouse secondary antibody (Jackson Immuno Research Labs, West Grove, PA). CD31 slides were washed free of unbound secondary in PBS and cover slipped for later imaging.

Pimonidazole and CD31 immunohistochemistry slides were both cover slipped with Vectashield fluorescence mounting medium (Vector Laboratories, Burlingame, CA). Slides for H&E were fixed in 4% PFA, rinsed and then subjected to a progressive staining line containing Leica Selectech reagents (Hematoxylin 560 and Alcoholic Eosin-Y 515) according to well established standard protocols. At the time of imaging, multiple 1600 × 1200 pixel subfields were acquired to comprise the entire tumor and surrounding tissue at 10x objective magnification on a Nikon E600 compound photomicroscope (Melville, NY) equipped with epi-fluorescence and bright-field illumination, an Applied Scientific Instruments x-y-z motorized stage (Eugene, OR) and Nikon DS-Fi2 CCD camera. Stage registry, illumination and camera were controlled by Nikon Imaging Solution Elements v4.20.00 software. Final scanned images ranging from 11844 × 10632 pixels to 16348 × 15330 pixels were parsed into registry and over-laid using Nikon Elements 4.20.00 and Adobe Photoshop CS4 (Adobe Systems, San Jose, CA).

## SUPPLEMENTARY MATERIALS FIGURES


