# Supplementary material for: Tumor physiological changes during hypofractionated stereotactic body radiation therapy assessed using multi-parametric magnetic resonance imaging
Source: Oncotarget. 2017 Mar 21;8(23):37464–77. doi: 10.18632/oncotarget.16395 (PMC5514922; doi:10.18632/oncotarget.16395)
Supplement: Supplementary file 1 [file oncotarget-08-37464-s001.pdf]

Tumor physiological changes during hypofractionated stereotactic body radiation therapy assessed using multi-parametric magnetic resonance imaging

SUPPLEMENTARY FIGURES

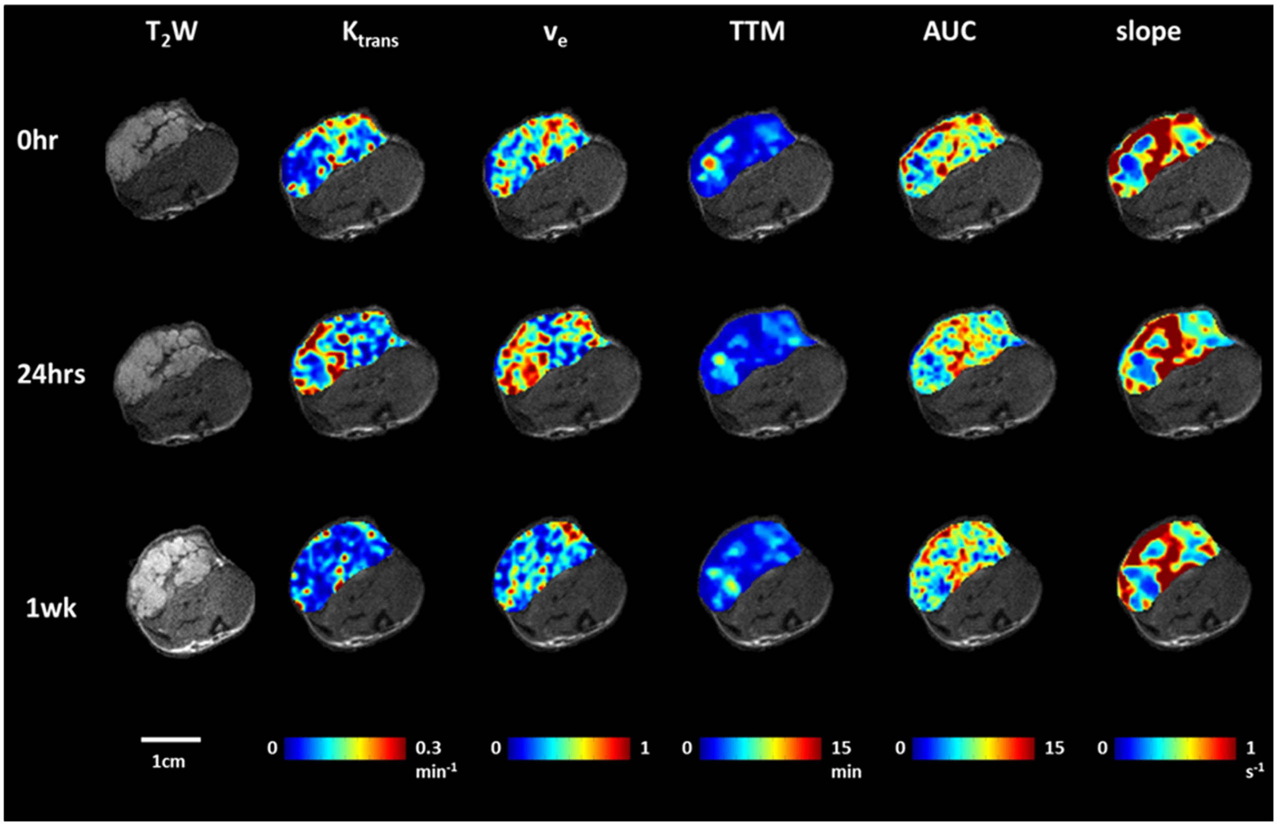

Supplementary Figure 1: DCE parametric maps of the representative control tumor that received no irradiation dose at baseline and 24 hours and 1 week later. T_2-weighted image and overlaid parametric response maps showing K^{trans}, v_e, TTM, AUC, and slope (left to right) of the same tumor as Figure 3. Distinct heterogeneity can be observed from all the functional maps at all the time points. No obvious changes were observed over one week.

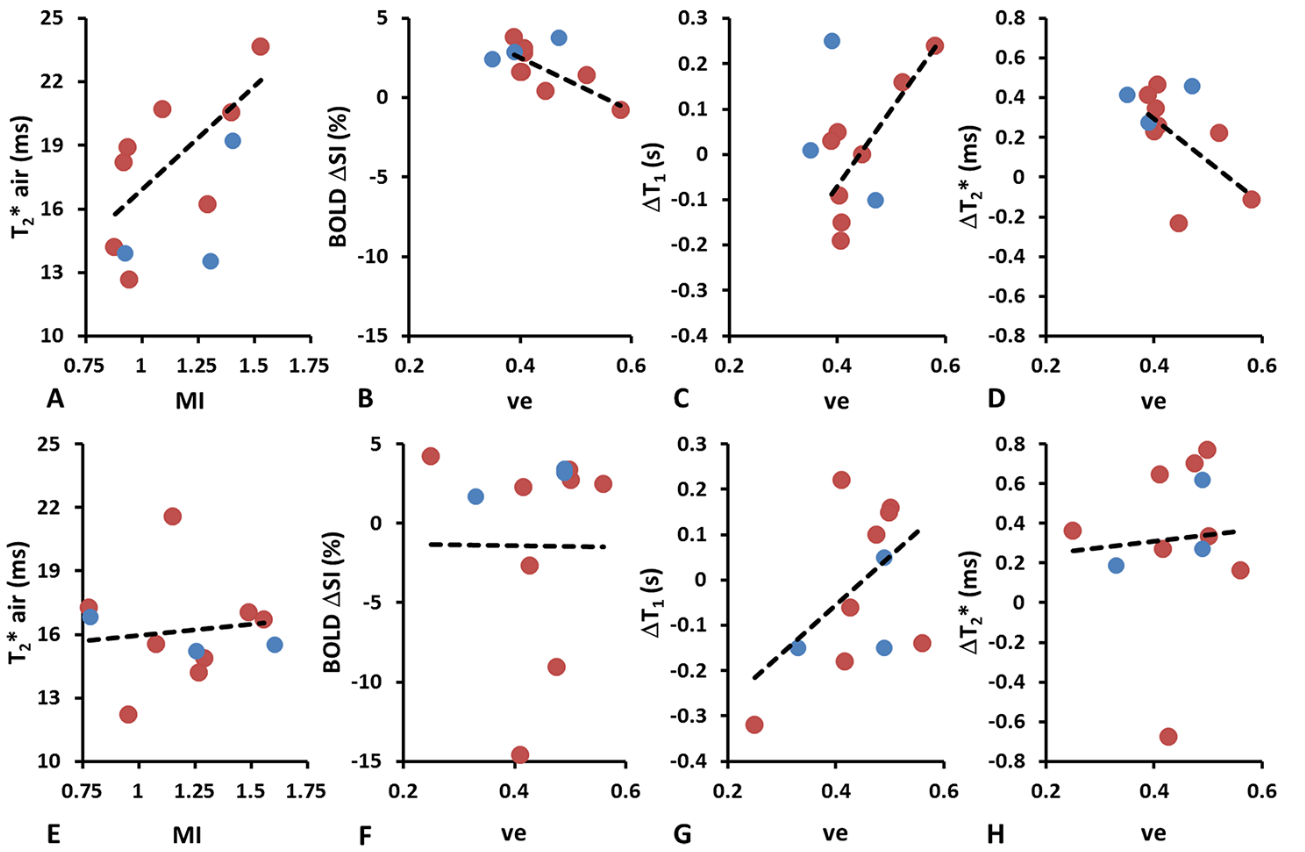

Supplementary Figure 2: Correlations between oxygen-sensitive MRI and DCE MRI. **(A-D)** baseline. **(E-H)** 24 hours. Each data point represents mean value for a tumor. MI is maximum signal intensity observed during DCE following IV infusion of Gadovist. V_e (the fraction of extravascular extracellular space). T_2*air is value during air breathing, while BOLD, ΔT_1 and ΔT_2* represent changes with respect to oxygen breathing challenge, *e.g.*, $\Delta T_1 = T_{1oxygen} - T_{1air}$. Red markers are data from Groups 2 and 3 (irradiated) and blue markers are data from Group 1 (control). Pearson correlation analysis was performed using irradiated tumor data only. From A to H, correlation coefficient R^2 = 0.45, 0.60, 0.64, 0.37, 0.01, 0.00003, 0.26 and 0.004.
